# Decision Trade-Offs in Ecological Momentary Assessments and Digital Wearables Uptake: Protocol for a Discrete Choice Experiment

**DOI:** 10.2196/47567

**Published:** 2023-09-25

**Authors:** Sherine El-Toukhy, James Russell Pike, Gabrielle Zuckerman, Phillip Hegeman

**Affiliations:** 1 Division of Intramural Research, National Institute on Minority Health and Health Disparities, National Institutes of Health Rockville, MD United States; 2 Department of Epidemiology, Bloomberg School of Public Health, Johns Hopkins University Baltimore, MD United States

**Keywords:** digital wearables, discrete choice experiment, ecological momentary assessment, mHealth, mobile health, remote monitoring technology

## Abstract

**Background:**

Ecological momentary assessments (EMAs) and digital wearables (DW) are commonly used remote monitoring technologies that capture real-time data in people’s natural environments. Real-time data are core to personalized medical care and intensively adaptive health interventions. The utility of such personalized care is contingent on user uptake and continued use of EMA and DW. Consequently, it is critical to understand user preferences that may increase the uptake of EMA and DW.

**Objective:**

The study aims to quantify users’ preferences of EMA and DW, examine variations in users’ preferences across demographic and behavioral subgroups, and assess the association between users’ preferences and intentions to use EMA and DW.

**Methods:**

We will administer 2 discrete choice experiments (DCEs) paired with self-report surveys on the internet to a total of 3260 US adults through Qualtrics. The first DCE will assess participants’ EMA preferences using a choice-based conjoint design that will ask participants to compare the relative importance of prompt frequency, number of questions per prompt, prompt type, health topic, and assessment duration. The second DCE will measure participants’ DW preferences using a maximum difference scaling design that will quantify the relative importance of device characteristics, effort expectancy, social influence, and facilitating technical, health care, and market factors. Hierarchical Bayesian multinomial logistic regression models will be used to generate subject-specific preference utilities. Preference utilities will be compared across demographic (ie, sex, age, race, and ethnicity) and behavioral (ie, substance use, physical activity, dietary behavior, and sleep duration) subgroups. Regression models will determine whether specific utilities are associated with attitudes toward or intentions to use EMA and DW. Mixture models will determine the associations of attitudes toward and intentions to use EMA and DW with latent profiles of user preferences.

**Results:**

The institutional review board approved the study on December 19, 2022. Data collection started on January 20, 2023, and concluded on May 4, 2023. Data analysis is currently underway.

**Conclusions:**

The study will provide evidence on users’ preferences of EMA and DW features that can improve initial uptake and potentially continued use of these remote monitoring tools. The sample size and composition allow for subgroup analysis by demographics and health behaviors and will provide evidence on associations between users’ preferences and intentions to uptake EMA and DW. Limitations include the cross-sectional nature of the study, which limits our ability to measure direct behavior. Rather, we capture behavioral intentions for EMA and DW uptake. The nonprobability sample limits the generalizability of the results and introduces self-selection bias related to the demographic and behavioral characteristics of participants who belong to web-based survey panels.

**International Registered Report Identifier (IRRID):**

DERR1-10.2196/47567

## Introduction

Ecological momentary assessments (EMAs) and digital wearables (DWs) are exemplars of remote monitoring technologies that afford us the ability to monitor the individual and their environment around the clock [1]. Remote monitoring technologies are a hallmark of precision medicine, whereby medical and health decisions can be tailored to the individual based on their unique and up-to-date data [2]. Specifically, EMA is an approach where people’s experiences and behaviors are repeatedly captured through brief self-report surveys [3]. DWs are electronic devices that repeatedly collect, process, store, and transmit data either directly through an internet connection or indirectly through a smartphone [4]. EMAs and DWs capture momentary contextual, sociopsychological, physiological, and behavioral data in the wild (ie, in real time and in a person’s natural environment), which improves the accuracy, granularity, and ecological validity of the measurements [5].

User-generated data on behavioral and medical conditions from EMAs and DWs can be used for diagnostic, management, and treatment purposes [1]. Examples include (1) detection of smoking and alcohol use episodes and lapses [6-8] and of various medical conditions [9] (eg, seizures [10] and atrial fibrillation) [11,12]; (2) management of diabetes [13], hypertension [14], physical activity, and diet [15-18]; and (3) treatment of cigarette smoking [19], sleep [20], and mood disorders [21] by intervening whenever and wherever support is needed. Data from EMAs and DWs can improve health and clinical outcomes through various mechanisms. EMAs and DWs capture events and behaviors, their determinants, and their effects. These data relate to several cognitive processes (eg, self-monitoring) underlying the self-regulatory mechanism that motivates and guides proactive and purposeful actions [22]. Additionally, data from EMAs and DWs represent feedback information to their users that is used to form and assess self-views and goals [23], thereby reinforcing desired behaviors [24]. Indeed, evidence shows that EMA and DW use is associated with improved health outcomes [25,26] (cf [27,28]).

Real-time EMA and DW data are the foundation of intensively adaptive health interventions and personalized medical care. Intensively adaptive interventions use ongoing information about the user to disseminate (or not) an appropriate treatment type and dose at the right time and place, relying on predefined decision rules that accommodate between- and within-user characteristics and other tailoring factors (eg, intrapersonal state and contextual cues) that change by the day, hour, or second over the course of an intervention [29,30]. For example, in Text2Quit, an interactive text messaging smoking cessation intervention, users’ data are periodically updated throughout the intervention (eg, number of cigarettes smoked) and subsequently integrated in pre- and postquit support messages [19]. In Sense2Stop, users wear chest and wrist DWs and answer smoking- and mood-related EMAs, both of which trigger digital prompts for users to engage in exercises to manage stress, a known antecedent of smoking lapse [31]. Although evidence on the efficacy and effectiveness of intensively adaptive interventions is largely based on pilot studies [32], they often outperform nonadaptive interventions [33]. Similarly, in clinical settings, continuous input from remote monitoring tools can be integrated into a patient’s electronic health record, which subsequently informs medical decision-making and improves clinical outcomes [34,35].

To reap the benefits associated with the use of remote monitoring technology, end users must uptake and continually use these digital tools. Uptake refers to the likelihood that an individual is willing to take part in an EMA or use a DW [36]. Continued use refers to the likelihood that an individual consistently completes the EMAs or uses DWs as prescribed [37]. Rates of EMA and DW uptake and continued use are frequently low or variable. For example, health and fitness mobile apps have a mere 3.7% retention rate 30 days post installation [38] and a third of DW owners stop using their devices within 6 months [39]. When reported, EMA completion rate varies across studies, between 20% and 90% in substance use studies [8] and between 44% and 96% in diet and physical activity studies [16]. Compounding these issues, the operationalization of uptake and continued use is often absent or inconsistent across research studies. For example, some studies report percentages of participants who engage in an activity or a previously set threshold of that activity, while others report averages or the exact number of times or number of days participants engage in a given activity [40]. Researchers have documented some of the facilitators of and barriers to the uptake and continued use of EMAs and DWs. Facilitators of use include perceptions of utility, usefulness, ease of use, usability, and having the motivation and ability to use the technology, to name a few [40]. Barriers include technical (eg, technology malfunction and incompatibility) and nontechnical (eg, digital literacy and cost) factors [40].

Uptake and continued use may be improved when the features of EMAs and DWs match the preferences of potential users. However, there is scarce evidence on the relative importance of different attributes and attribute levels of EMAs and DWs that might affect uptake and potentially continued use, although uptake and continued use are driven by different factors [4]. Indeed, the design of EMAs varies greatly regarding key attributes. For instance, the length of EMA studies ranges from 1 to 182 days, with a median of 7 days, whereas the number of daily prompts ranges from 1 to 42 [41]. Additionally, user preferences for specific attributes are rarely examined across sociodemographic and behavioral subgroups, despite well-documented differences in uptake and continued use of remote monitoring technologies between these groups [41,42]. There is also little evidence on the relationship between EMA and DW preferred attributes and intentions to use remote monitoring technologies.

Using a discrete choice experiment (DCE) [43], this study aims to identify the optimal attributes of EMAs and DWs that may increase uptake and continued use. A DCE is a quantitative survey-based methodology that elicits preferences by presenting participants with alternatives and asking them to make a choice. The process is repeated with different combinations of attributes, and the resulting data are used to calculate the relative importance each participant places on each attribute [44]. In previous studies, this method has been used to elicit preferences for health mobile apps [45-47], sharing health data [48,49], and health interventions [50]. In this study, 2 DCEs will elucidate preferences for EMAs and DWs, examine how these preferences vary across demographics (ie, sex, age, and race and ethnicity) and behaviors (ie, substance use, physical activity, dietary behavior, and sleep duration), and assess whether specific preferences are associated with attitudes toward or intentions to use EMAs and DWs in the future.

## Methods

### Aims

This study aims to (1) quantify the relative importance of 5 EMA attributes (ie, prompt frequency for surveys, number of questions per prompt, prompt type, health topic, and assessment duration) and 30 DW features centered around 6 domains (ie, device characteristics, effort expectancy, social influence, and facilitating conditions related to technical, health care, and market factors); (2) identify the relative importance of EMA and DW attributes within demographic (ie, sex, age, and race and ethnicity) and behavioral (ie, those who meet vs who do not meet predefined criteria or guidelines for a given behavior) subgroups; and (3) examine the associations between users’ preferences and EMA and DW uptake intentions.

### Design

The focal point of the study is 2 DCEs (Multimedia Appendix 1). Each participant will complete only 1 DCE.

The first DCE will assess EMA preferences using a choice-based conjoint design [51,52] in which 5 attributes (eg, number of surveys per day) are presented as a package. Each attribute is assigned one of several levels (eg, 2-3 surveys or 6 or more surveys). Participants choose between 2 packages or neither package. Attributes and levels (Table 1) were selected from previous research on EMAs [15,16,18,41,53]. Collectively, over 1500 unique packages can be constructed from these attribute levels. However, a balanced fractional factorial design will be implemented in Qualtrics so that each participant only needs to repeat the task 9 times and evaluate 18 packages.

The second DCE will measure DW preferences using a maximum difference scaling design [54,55] in which participants view a subset of 4 features from a larger set of 30 features (Table 2). Participants choose which feature in the subset they value the most and which they value the least. The attributes and corresponding statements were adapted and developed from previous research on DWs [4,56-60] and digital health tools more broadly [40,46,48,50,61-65]. Attribute domains correspond to constructs derived from technology acceptance theoretical frameworks [66]. The attributes and corresponding statements center around effort expectancy, defined as the level of ease in using the technology; social influence, defined as the level of support the user of the technology receives from important others; and facilitating conditions related to technical infrastructure, health care, and market factors that can prohibit or support the technology use [66]. We also include device characteristics as an external factor associated with technology acceptance [59]. Although over 24,000 combinations of attributes are possible, each participant will only be required to complete the task 23 times.

**Table 1:** Attributes and attribute levels in an ecological momentary assessment discrete choice experiment.

Attributes and attribute levels		Descriptions
**Prompt frequency**		
	0-1 per day	None or only 1 survey
	2-3 per day	2-3 surveys
	4-5 per day	4-5 surveys
	≥6 per day	6 or more surveys
**Number of questions per prompt**		
	1 question	1 question
	2-3 questions	2-3 questions
	4-5 questions	4-5 questions
	≥6 questions	6 or more questions
**Prompt type**		
	Event-contingent	Self-initiated when a predefined event has occurred (eg, smoking a cigarette, snacking between meals, or being in a specific location such as a bar)
	Signal-contingent	At random times
	Time-contingent	On fixed times
	Mixed	Combination of random and fixed times
**Health topic**		
	Nicotine or tobacco use	Nicotine or tobacco use
	Alcohol use	Alcohol drinking
	Marijuana use	Marijuana use
	Physical activity	Exercise
	Nutrition	Diet or nutrition
	Sleep	Sleep
**Assessment duration**		
	≤1 month	1 month or shorter
	>1 but <6 months	More than 1 month but less than 6 months
	≥6 but <12 months	More than 6 months but less than 1 year
	≥1 year	1 year or longer

**Table 2:** Attributes examined in the digital wearables discrete choice experiment.

Domains and attributes		Statements	
**Device characteristics**			
	Type	The device collects nonmedical data (eg, steps) The device collects medical data (eg, blood glucose)	
	Point of contact	I must wear the device on my chest or ankle	
	Battery life	The device must be charged every 48 hours	
	Display	The device has a touchscreen interface	
	Memory	The device has a memory chip to prevent data loss	
	Cellular data	The device must be always connected to the internet	
	Personalization	I can personalize the device to my individual goals and preferences I can change the look and feel of the device (eg, by using different strap styles and colors)	
	Added features	The device provides information in languages other than English (eg, Spanish) The device allows me to interact with other users if I want to	
	Effectiveness	The accuracy of the device has been proven in scientific studies	
**Effort expectancy**			
	Ease of use	The device is easy to use	
	Data sync	The device syncs automatically with a smartphone	
	Comfort	The device fits with my lifestyle and daily activities The device can cause skin irritations	
**Social influence**			
	Peer recommendation	A friend or family member recommended the device to me	
**Facilitating technical factors**			
	Compatibility	The device is compatible with all smartphones (eg, Android and Windows) The device is compatible with popular health apps	
	Data security and privacy	I can enable or disable location tracking on the device I get to choose when and how the device shares data My data are encrypted My data will be sold to third parties for profit	
**Facilitating health care factors**			
	Insurance	The device is covered by health insurance	
	Doctor recommendation	My doctor recommended the device to me	
	Data exchange	I can share the data with my doctor	
**Facilitating market factors**			
	Costs	The device is under US $150	
	Customer reviews	The device has a high customer rating	
	Warranty	The device comes with a 2-year warranty	
	Customer service	There is a number I can call if the device stops working	

### Sample

A nonprobability sample of 3260 US adults, 18 years and older, will be recruited nationwide to participate in a web-based survey. The sample will be equally split across biological sex, age groups, and race and ethnicity to allow comparisons of users’ preferences within demographic subgroups (Table 3). Sample size calculations were performed using standard DCE formulas [67,68]. A sample of 333 participants is required for the EMA DCE based on the formula n≥1000(x)/yz, where *x* is the maximum number of levels of any attribute, *y* is the number of choices per task excluding the option none, and *z* is the number of times the task is completed per participant [ie, 1000(6)/(2 × 9) = 333]. A sample of 163 participants is required for the DW DCE based on the formula n≥500(w)/yz, where *w* is the number of features and *y* and *z* are defined identically to the previous formula [ie, 500(30)/(4 × 23) = 163]. However, the minimum sample for the DW DCE will be set at 300 participants in accordance with recommended guidelines [68]. Based on these calculations, the study should be well powered even when the sample is further stratified by demographic and behavioral subgroups.

**Table 3:** Target sample characteristics (N=3260).

Characteristics		Participants, n (%)
**Sex**		
	Female	1630 (50)
	Male	1630 (50)
**Age (years)**		
	18-29	815 (25)
	30-44	815 (25)
	45-59	815 (25)
	≥60	815 (25)
**Race and ethnicity**		
	Non-Hispanic American Indian, Alaska Native, Native Hawaiian, or Pacific Islander	652 (20)
	Non-Hispanic Asian	652 (20)
	Hispanic or Latino	652 (20)
	Non-Hispanic Black	652 (20)
	Non-Hispanic White	652 (20)

### Recruitment

Through its research panels, Qualtrics will send an invitation email to individuals with demographic characteristics that match the target sample. The email includes links to 2 identical surveys: the first has the EMA DCE, and the second has the DW DCE. The email also includes an estimated time to complete the survey and the incentive offered. Qualtrics will compensate participants and distribute the incentives at a rate equivalent to surveys of similar scope, burden, and duration. Data collection will continue until the desired sample size and composition are reached.

### Procedures

Participants will consent before proceeding to any survey questions. Consenting participants will respond to inclusion and exclusion questions. To be included in the study, participants must be adults residing in the United States. Inclusion questions include zip code, age, biological sex, and race and ethnicity. The latter 3 questions are the basis of built-in hidden quotas for sample characteristics (Table 3). When a quota is met (eg, 50% females already recruited), participants who belong to that subgroup will not be allowed to take the survey. The only exclusion criterion is when participants respond yes to “Do you use any fitness tracker, smartwatch, or electronic medical device to monitor or track your health?” [69]. Participants who use a DW are excluded to improve inferences about preferences among nonusers. Ineligible participants receive a thank you message and are not allowed to proceed any further. Eligible participants will proceed to the remainder of the study questions. Except for completing 1 of the 2 DCEs, all instructions and questions are identical for all participants. The survey will take about 30 minutes to complete.

### Measures

We will collect sociodemographic (ie, zip code, biological sex, age, race and ethnicity, sexual orientation, education, household income, employment, marital status, and English language proficiency) [69,70] and health (ie, weight and height [69], general health [71], emotional health [72], perceived susceptibility to disease [73], underlying health conditions [70], having a regular health care provider [69], and health insurance [69]) data. We also collect behavioral data on nicotine and tobacco use [74,75], marijuana use [76], alcohol use [77], fruit and vegetable intake [69], physical activity [69], and sleep [69], all of which are risk factors for chronic conditions and are thus the focus of health and medical interventions. These behavioral data allow comparisons of users’ preferences within behavioral subgroups. Where each participant can demonstrate any number of the 6 behaviors examined in the study, we follow public health guidelines to group each participant into 1 of 2 groups, one where participants do (vs do not) meet those guidelines and thresholds for each behavior as follows: (1) Nicotine and tobacco use is defined as current use of any nicotine or tobacco product [78]. (2) Marijuana use is defined as current use of marijuana or cannabis [78]. (3) Alcohol misuse is defined as daily consumption of >1 drink per day for women and >2 drinks per day for men, or as total consumption of >7 drinks per week for women and >14 drinks per week for men [79,80]. Alcohol use questions are asked only of participants 21 years or older. (4) Insufficient physical activity is defined as <150 minutes or 2.5 hours of physical activity per week [81]. (5) Inadequate fruit and vegetable intake is defined as <5 servings of fruit and vegetables total per day [79]. (6) Insufficient sleep is defined as <7 hours of sleep per night [82].

We also collect data on digital technologies access and use [69,83]; phone affinity [84]; previous use of [69], attitudes toward [85], and satisfaction with health applications [86]; attitudes toward wearable devices [85]; technology acceptance [59,87-89]; and willingness and intentions to use EMAs and DWs.

### Data Analysis

Data from both DCEs will be analyzed using hierarchical Bayesian multinomial logistic regression models [90,91]. These models will generate subject-specific utilities quantifying the relative importance of each attribute to each participant. EMA and DW attribute preferences will be characterized among all participants and within subgroups defined by sex (female or male), age group (18-29 years, 30-44 years, 45-59 years, or 60 years or older), race and ethnicity (Non-Hispanic American Indian, Alaska Native, Native Hawaiian, or Pacific Islander; Non-Hispanic Asian; Hispanic or Latino; Non-Hispanic Black; or Non-Hispanic White), or health behavior (meeting vs not meeting established public health guidelines for each behavior). Differences between subgroups will be evaluated using *t* tests, analysis of variance, or linear regression, as appropriate. The association between each utility and each measure of attitude toward or intention to use EMAs and DWs will be depicted in correlation matrices generated from data comprising all participants or data from specific demographic or behavioral subgroups. A latent profile analysis [92,93] of individual-level utilities will be performed to detect segments of individuals who share common preferences. The extent to which demographics or behavioral characteristics predict membership in these segments will be quantified using the 3-step method [94,95]. The Bolck, Croon, and Hagenaars method [96] will evaluate whether specific segments are associated with specific attitudes or intentions.

### Ethical Considerations

The study was deemed exempt on December 19, 2022, under category 2: research that only includes interactions involving educational tests, survey procedures, interview procedures, or observation of public behavior [§45 CFR 46.10(d)(2)], National Institutes of Health Institutional Review Board (001208).

## Results

Data collection started on January 1, 2023, and concluded on May 4, 2023. Data analysis is ongoing.

## Discussion

This protocol outlines a survey-based DCE to identify attributes that are associated with the uptake of EMAs and DWs. EMAs and DWs are remote monitoring technologies that capture real-time data in users’ natural environments that are foundational to precision medicine. These data-driven approaches to health and medical care are the face of an increasingly digital ecosystem and align with the emphasis on person-centered care [97].

The study will generate insights on the optimal attributes and features that users value to maximize the uptake of EMAs and DWs. Such insights have significant health and medical implications given the ubiquitousness of mobile technologies. In 2021, smartphone ownership was at 85% in the United States [98] and there were approximately 100,000 health care apps on the Apple App Store and Google Play Store [99,100]. As of 2022, wearables penetration was at 25.3% among US adults [101] and is forecast to reach 628.3 million devices globally by 2026 [102]. Additionally, mobile health technologies enjoy acceptance and demand from users and patients [103]; interest of commercial, research, and health care entities [104,105]; and support of national and international policies and initiatives [106,107]. These technologies have given rise to terms like digital biomarkers, digital diagnostics, digital therapeutics, and digital treatments [1]. They are becoming common in medical research as well as available directly to users who can engage in self-care within (eg, electronic health records) [108] or outside (eg, faith organizations) [109] the traditional health care system. This is especially relevant given the prevalence of chronic conditions (eg, heart disease) and associated modifiable risk factors (eg, tobacco use) that are suited for remote monitoring [110]. Programs for the integration of telemonitoring in clinical and medical care for managing and treating chronic conditions in patients are developed and piloted [26,34], providing a far-reaching platform for health care delivery that can benefit underserved populations such as those with limited access to health care or those who disproportionally bear the highest burden of disease and risk factors [111].

The study has several limitations. Participants belong to web-based survey panels, rendering the results nongeneralizable to the US population and raising concerns about self-selection bias. The Qualtrics platform does not permit the inclusion of 2 DCEs in the same survey. Accordingly, participants received an invitation email with links to both surveys but were permitted to take only one. Although current use of a wearable device is an exclusion criterion for this study, the sample can include ex-users of wearables who may hold preexisting attitudes toward DWs based on previous experiences. Because of the cross-sectional nature of the survey, we capture behavioral intentions rather than EMA and DW use behavior. To avoid survey burden, we limited the number of EMA attributes and DW features. The study has several strengths, including a robust sample size that allows for subgroup analysis by sociodemographic and behavioral groups. The study also examines the preferences-intentions relationship that provides actionable information to increase the uptake of EMAs and DWs, especially among at-risk or disadvantaged populations.

## Appendix

Multimedia Appendix 1 Discrete choice experiment tasks.

## Abbreviations

DCE: discrete choice experiment
DW: digital wearable
EMA: ecological momentary assessment

## Data Availability

Data sharing is not applicable to this article as no data sets were generated or analyzed during this study.
