# Supplementary material for: Decision Trade-Offs in Ecological Momentary Assessments and Digital Wearables Uptake: Protocol for a Discrete Choice Experiment
Source: JMIR Res Protoc. 2023 Sep 25;12:e47567. doi: 10.2196/47567 (PMC10562974; doi:10.2196/47567)
Supplement: Multimedia Appendix 1 [file resprot_v12i1e47567_app1.docx]

**Exhibit 1.** Discrete choice experiment tasks.

| **Experiment 1: Ecological momentary assessment**  *If you were asked to answer a brief survey with the following sets of characteristics, which one you would be most likely to complete?*   \| **Number of surveys per day** \| \| 2 to 3 surveys \| 6 or more surveys \| None. I would not choose either of these. \| \| --- \| --- \| --- \| --- \| --- \| \| **Number of questions in each survey** \| \| 6 or more questions \| 1 question \| \| **Timing of survey** \| \| At random times \| At fixed times \| \| **Topic of survey** \| \| Exercise \| Nicotine or tobacco use \| \| **Length of assessment** \| \| 1 month or shorter \| One year or longer \| \|  \| **[Choice 1]** \| \| **[Choice 2]** \| **[Choice 3]** \| |
| --- | --- | --- | --- | --- | --- | --- | --- | --- | --- | --- | --- | --- | --- | --- | --- | --- | --- | --- | --- | --- | --- | --- | --- | --- | --- | --- |
|  |
| **Experiment 2: Digital wearables**  *Choose the reason for using a wearable device that is most important to you and least important to you.*   \| **Most** \|  \| **Least** \| \| --- \| --- \| --- \| \|  \| The device must be charged every 48 hours \|  \| \|  \| I can enable or disable location tracking on the device \|  \| \|  \| The device is covered by health insurance \|  \| \|  \| The device is under $150 \|  \| |
